# Supplementary material for: Cryo EM structure of intact rotary H+-ATPase/synthase from Thermus thermophilus
Source: Nat Commun. 2018 Jan 8;9:89. doi: 10.1038/s41467-017-02553-6 (PMC5758568; doi:10.1038/s41467-017-02553-6)
Supplement: Supplementary file 1 — Supplementary Information [file 41467_2017_2553_MOESM1_ESM.pdf]

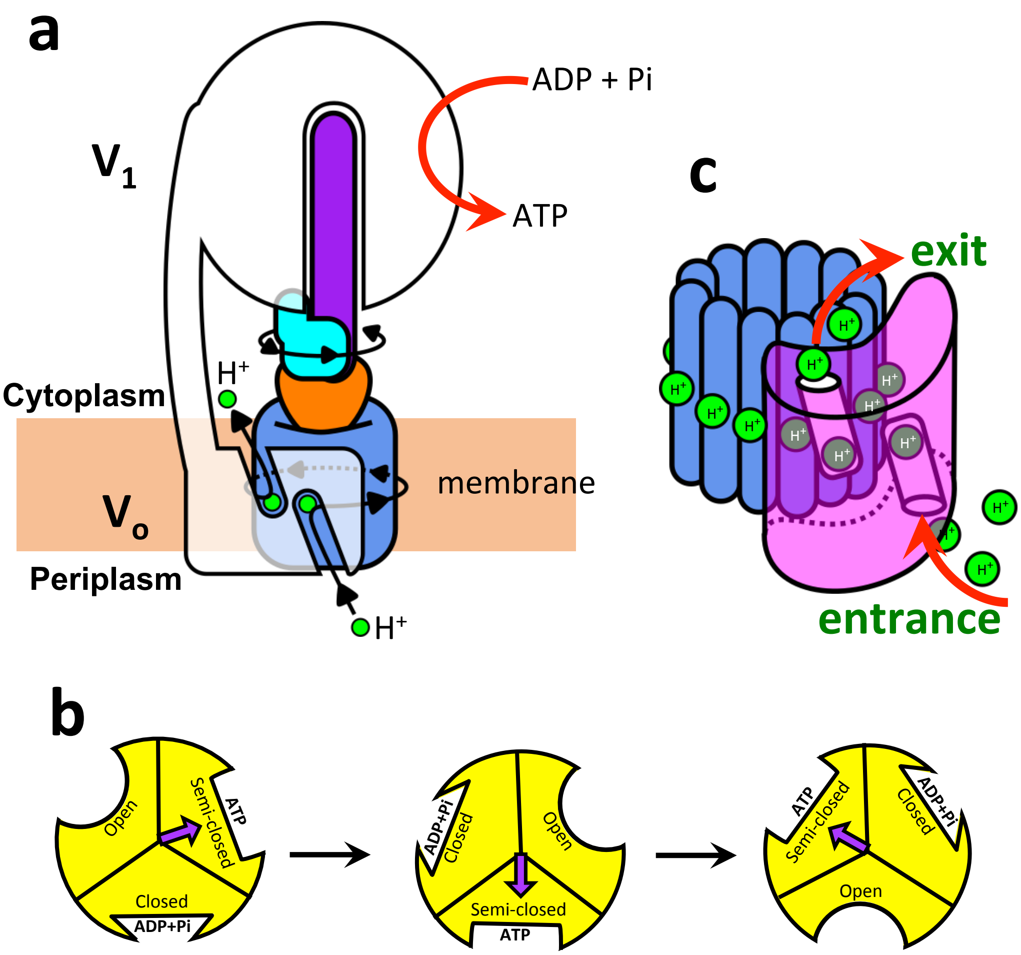

Supplementary figure 1. Schematic model for rotary ATPase and the mechanism.
a. Structure of rotary ATPase. The rotary ATPase is a reversible molecular motor. The ATPase catalyzes proton translocation across the membrane when ATP hydrolysis in A_3B_3 rotates the central rotor complex. The surrounding stator apparatus (white) synthesizes ATP from ADP and inorganic phosphate by rotation of the central rotor (colored) driven by the electrochemical potential across the membrane. **b**. The three catalytic sites in A_3B_3. These adopt three different nucleotide binding conformations, open, closed, and semi-closed. The ATPase domain changes three states of binding site substantially, resulting in rotation of the central rotor (purple arrow). Unlike F_1, the V_1 does not show an apparent substep in the ATPase mode. **c**. Two channel model for H^+-rotary ATPase. Proton translocation occurrs through the periplasmic and cytoplasmic pores colored in transparent magenta. In the *T. thermophilus* V/A-ATPase, one rotation of the rotor ring translocates 12 protons.

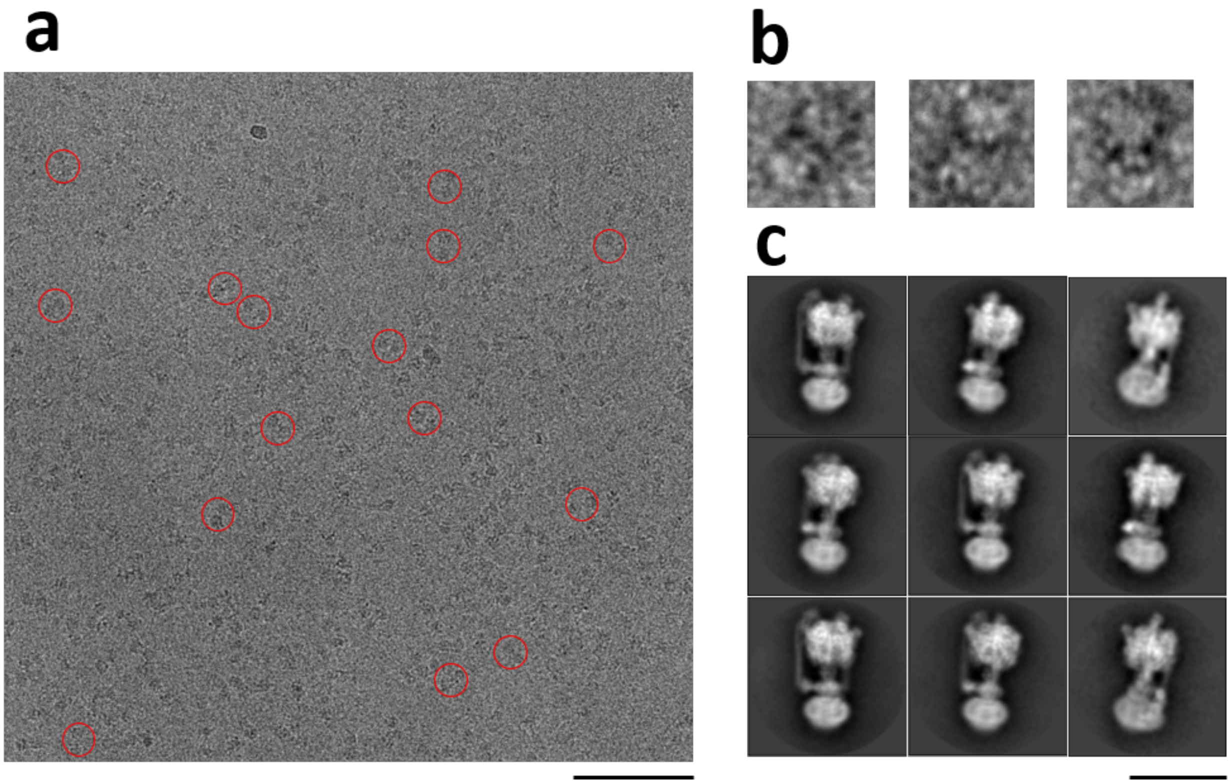

Supplementary figure 2. ***Tth*** **V/A-ATPase particle images. a**, Example micrograph with *Tth* V/A-ATPase particles circled in red. Scale bar, 1 μm. **b**, Representative V/A-ATPase single particles obtained from manual picking. **c**, Example of 2D class averaged images from all particles obtained from reference-free classification. Scale bar, 200 Å.

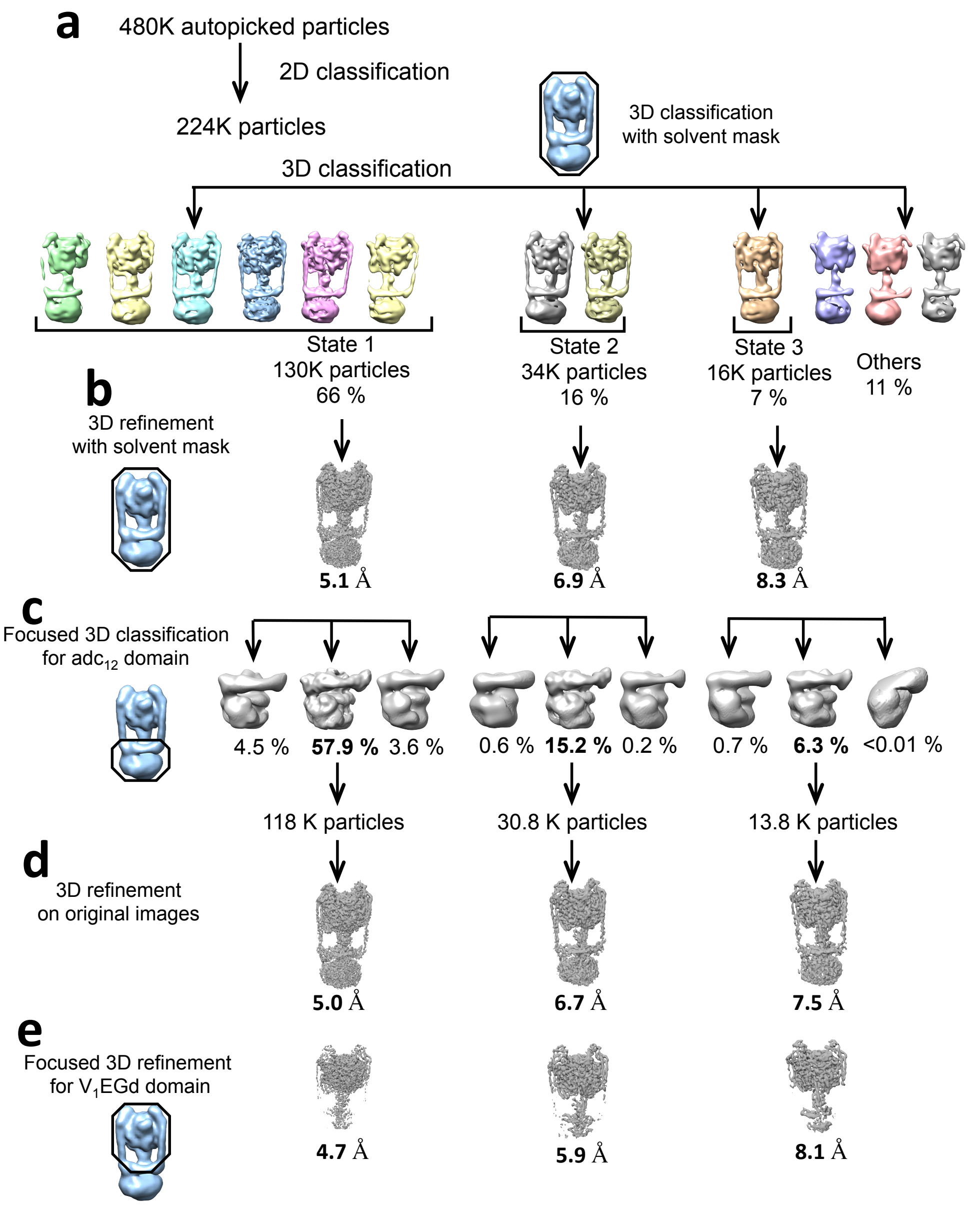

Supplementary figure 3. Image processing procedures for V/A-ATPase single particle analysis. All processing steps were analyzed by RELION2.0. Representative micrograph and representative 2D class averages from reference-free 2D classification are shown in Supplementary figure 1. **a**, The ~224k particles were used for 3D classification into 12 classes by imposing a specific mask cutting into protein density. **b**, Resulting classes were further classified by eye based on orientation of the F subunit, and the classes with the same orientation were combined. **c-e**, Focused 3D classification and refinement approaches for both the whole ATPase (**c**, **d**) and the hydrophilic V_1EGd (**e**, $A_3B_3DF(EG_{CT})_2d$) subcomplex only gave improved maps for each state. All resolutions are based on the gold-standard Fourier shell correlation (FSC) =0.143 criterion.

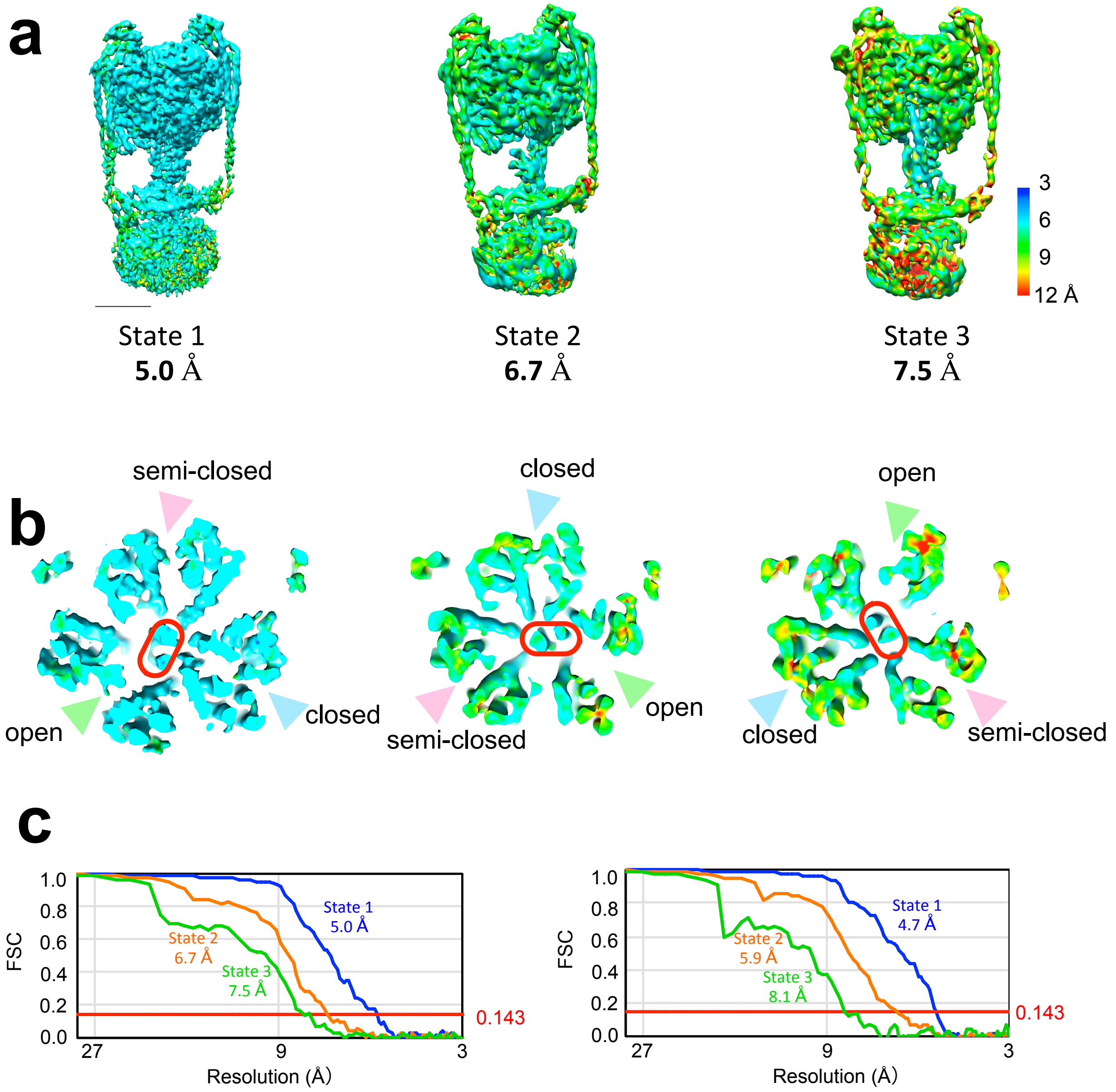

Supplementary figure 4. Local resolution of the three maps. **a**, Local resolution estimation by Resmap for *Tth* V/A-ATPase maps. Scale bar, 50 Å. Horizontal sections through V_1 are shown in **b**. **c**, Fourier shell correlation (FSC) curve for the three *Tth* V/A-ATPase maps (left) and V_1EGd ($A_3B_3DF(EG_{CT})_2d$) maps (right). The overall resolution of the map at FSC = 0.143 is indicated by the red line.

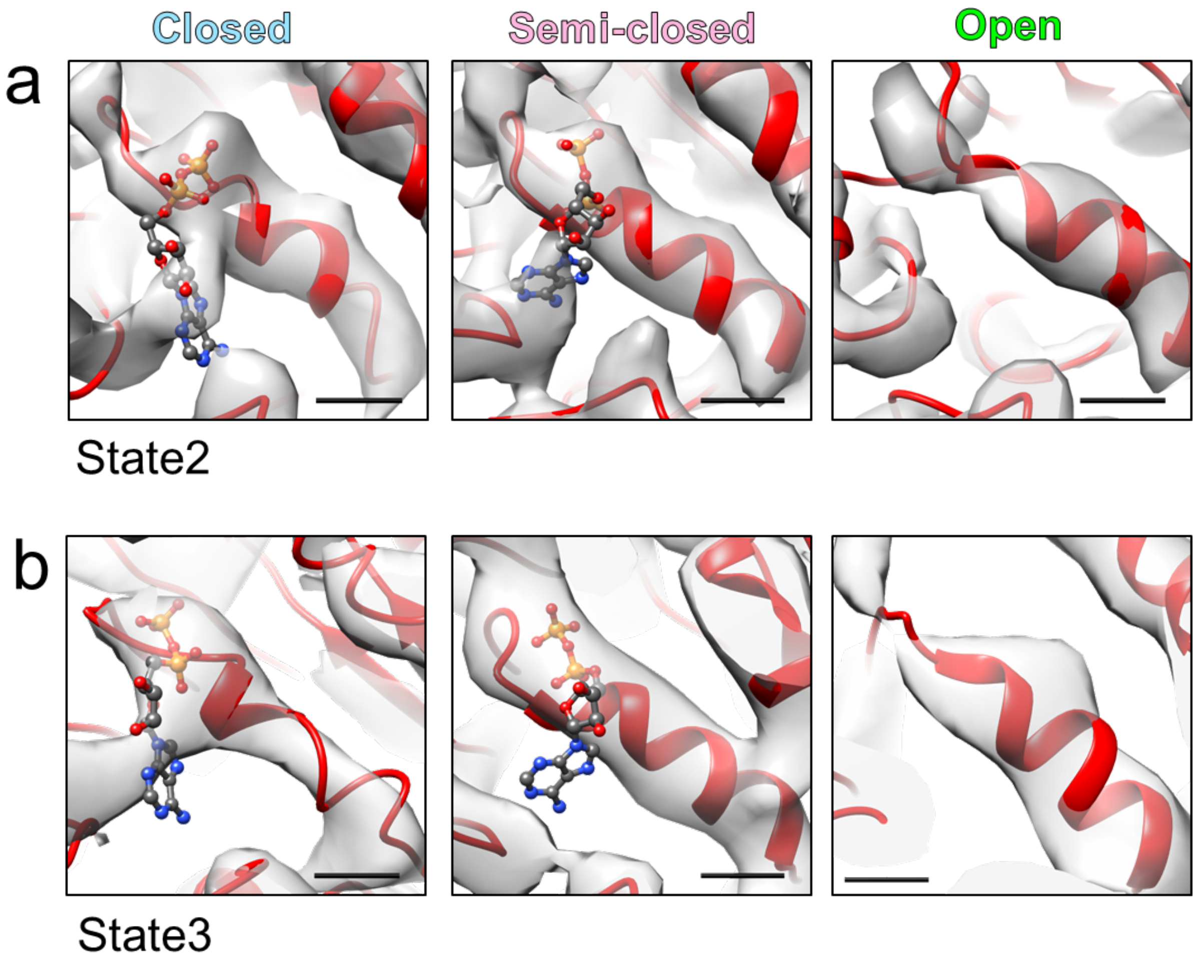

Supplementary figure 5. Magnified views of the nucleotide-binding sites in the AB pairs in state2 (a) and 3 (b). ADP is depicted in and ball-and-sticks format. Experimental maps, V_1EGd of state2 and VoV1 of state3 (Supplementary Fig.3d-e), are shown in semi-transparent grey. Scale bar, 5 Å.

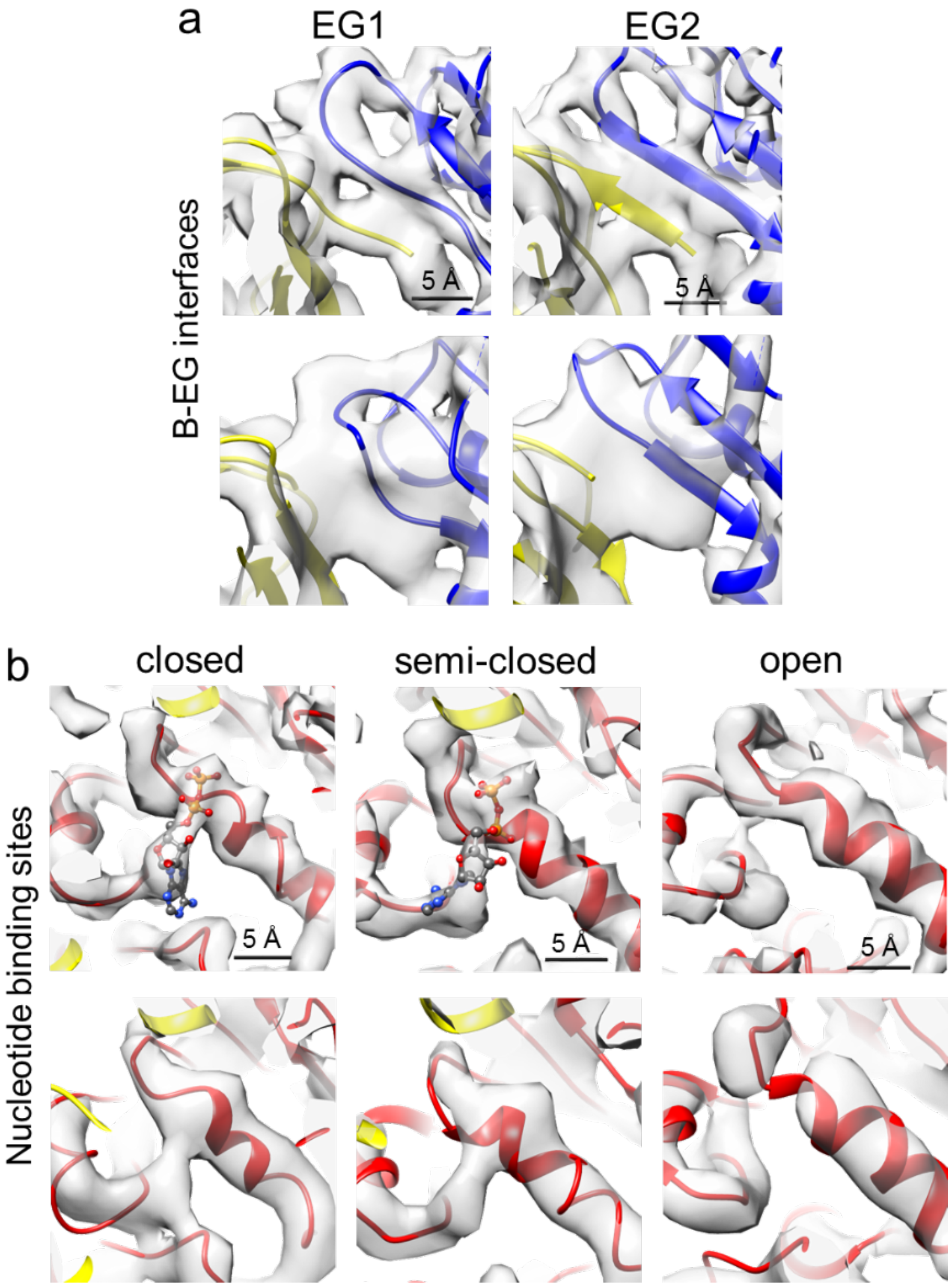

Supplementary figure 6. Comparison of the EM map of state1 of the V/A-ATPase produced as part of this study (upper panels) with that of the previous EM map by Schep *et al.* (lower panels).

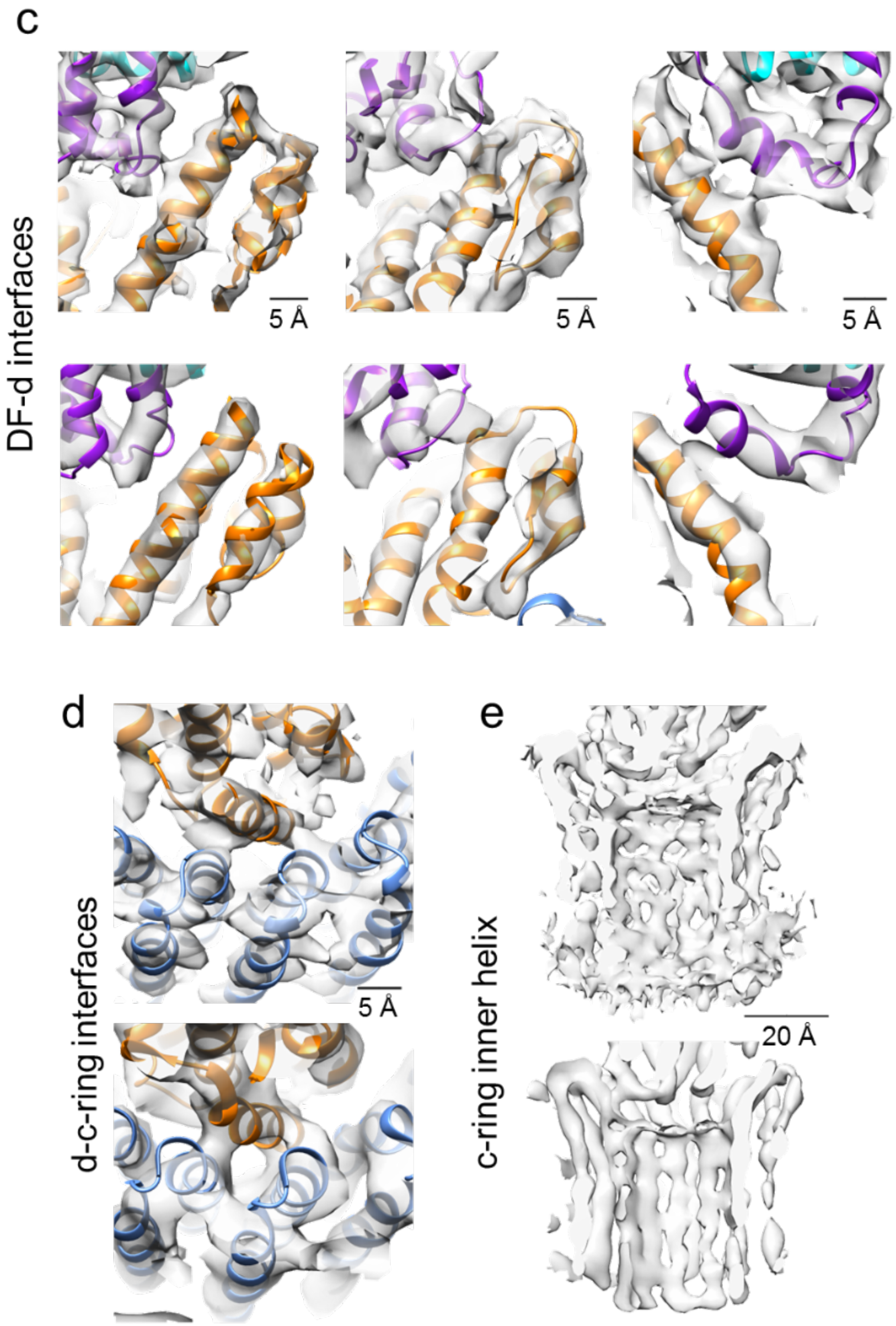

Supplementary figure 6. Comparison of the EM map of state1 of the V/A-ATPase produced as part of this study (upper panels) with that of the previous EM map by Schep *et al.* (lower panels).

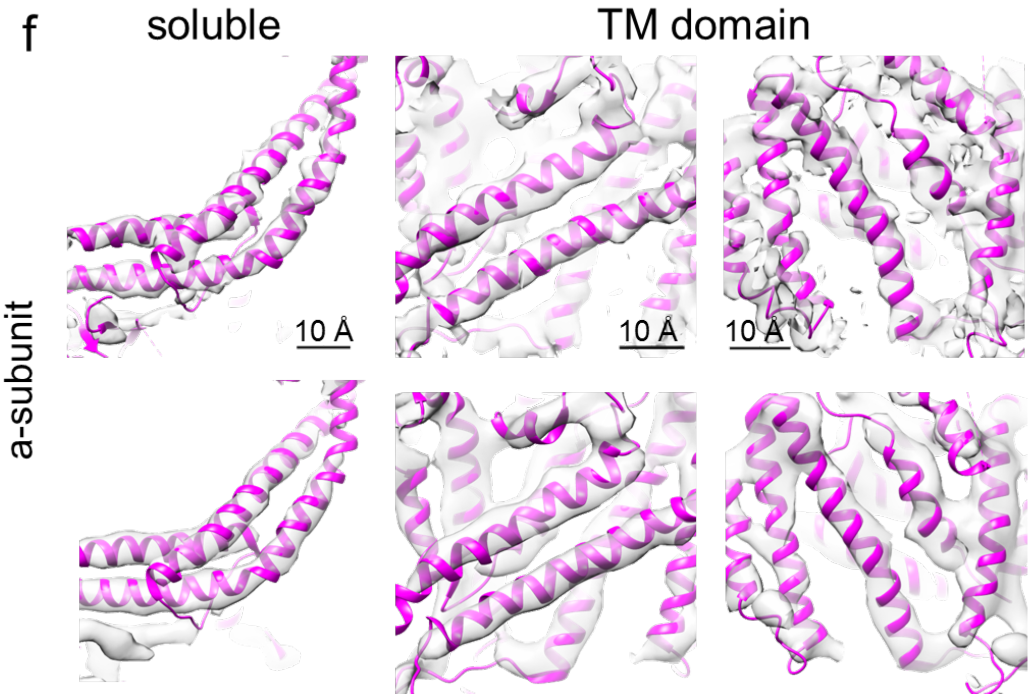

Supplementary figure 6. Comparison of the EM map of state1 of the V/A-ATPase produced as part of this study (upper panels) with that of the previous EM map by Schep *et al.* (lower panels).

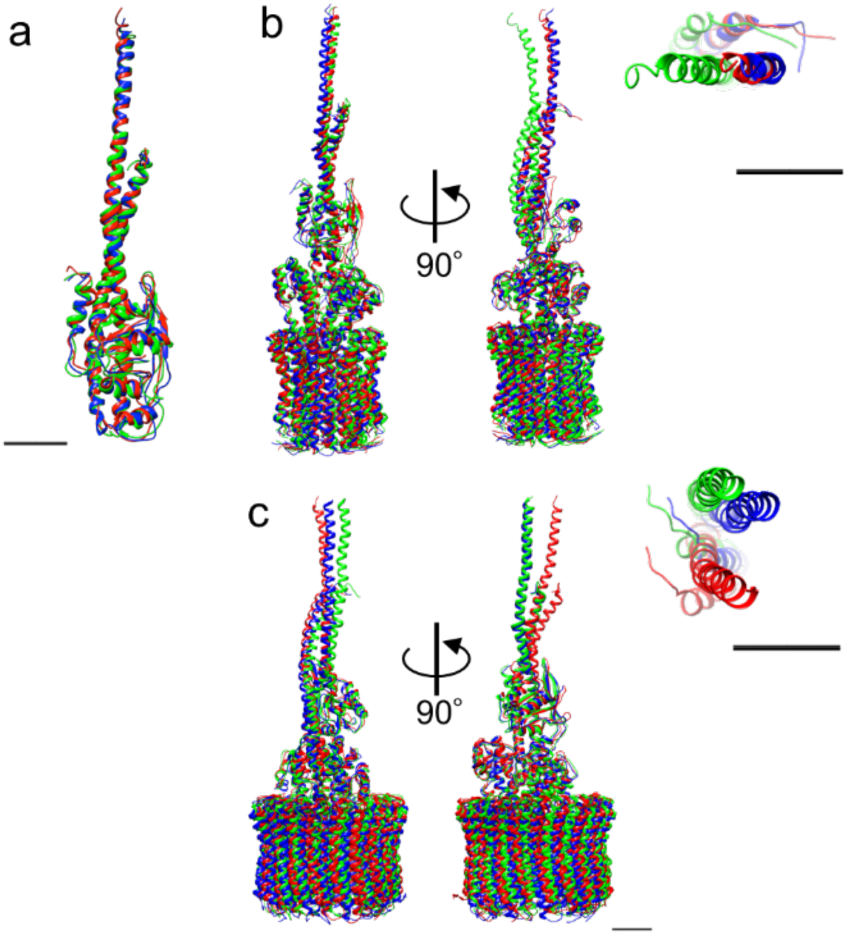

Supplementary figure 7. Structural comparison of the rotor complex in state1-3. **a**, Side views of the models of the D and F subunits in each state when these subunits are superimposed at the D subunit. State1, 2 and 3 are colored red, blue and green, respectively. **b**, Structural comparison of the helical region of the rotor complex when superimposing the d subunit in state1-3 of the V/A-ATPase. **c.** Structural comparison of the helical region of rotor complex when superimposing d subunit in state1-3 of the yeast V-ATPase (PDBID: 3J9T, 3J9U, and 3J9V). Scale bar, 20 Å.

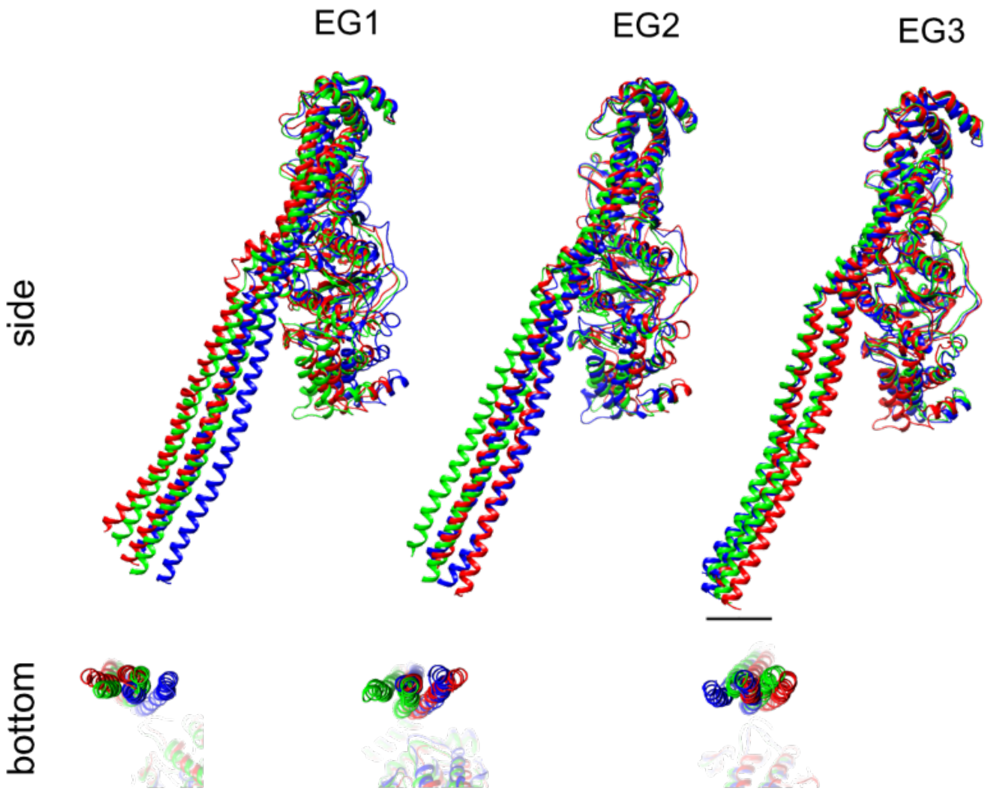

Supplementary figure 8. Structural comparison of the EG stalk when superimposing β barrel of B subunit in state1-3 of yeast V-ATPase (PDBID: 3J9T, 3J9U, and 3J9V). Scale bar, 20 Å.

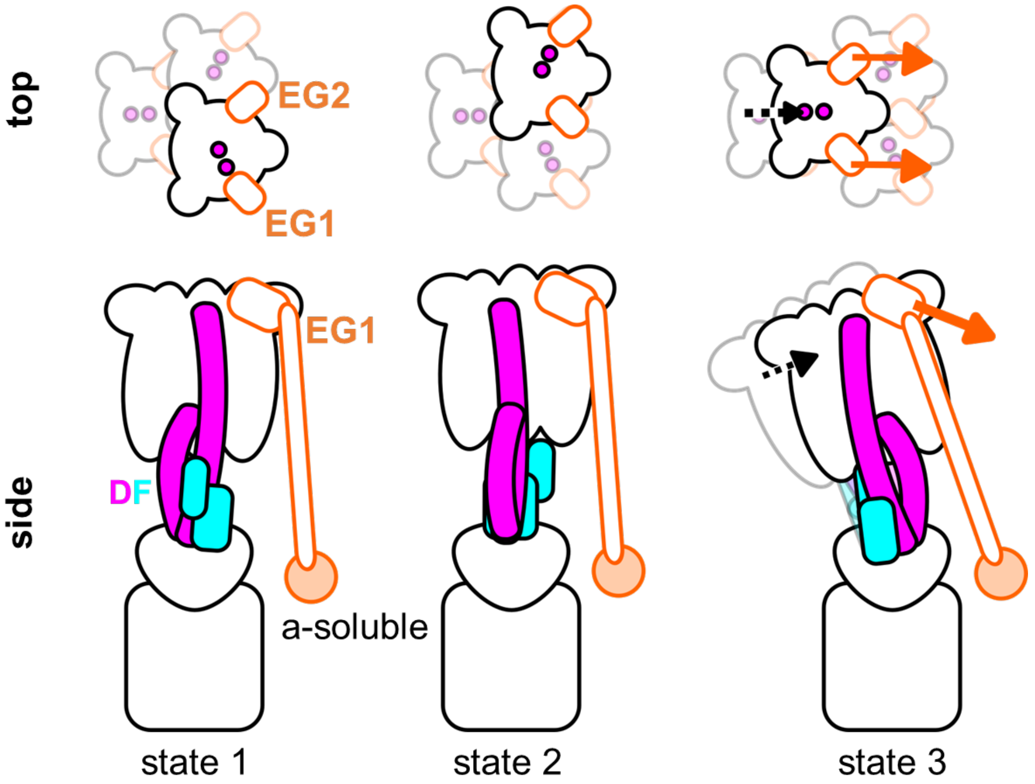

Supplementary figure 9. Schematic model for the molecular basis of different conformation of state3 of the V/A-ATPase.

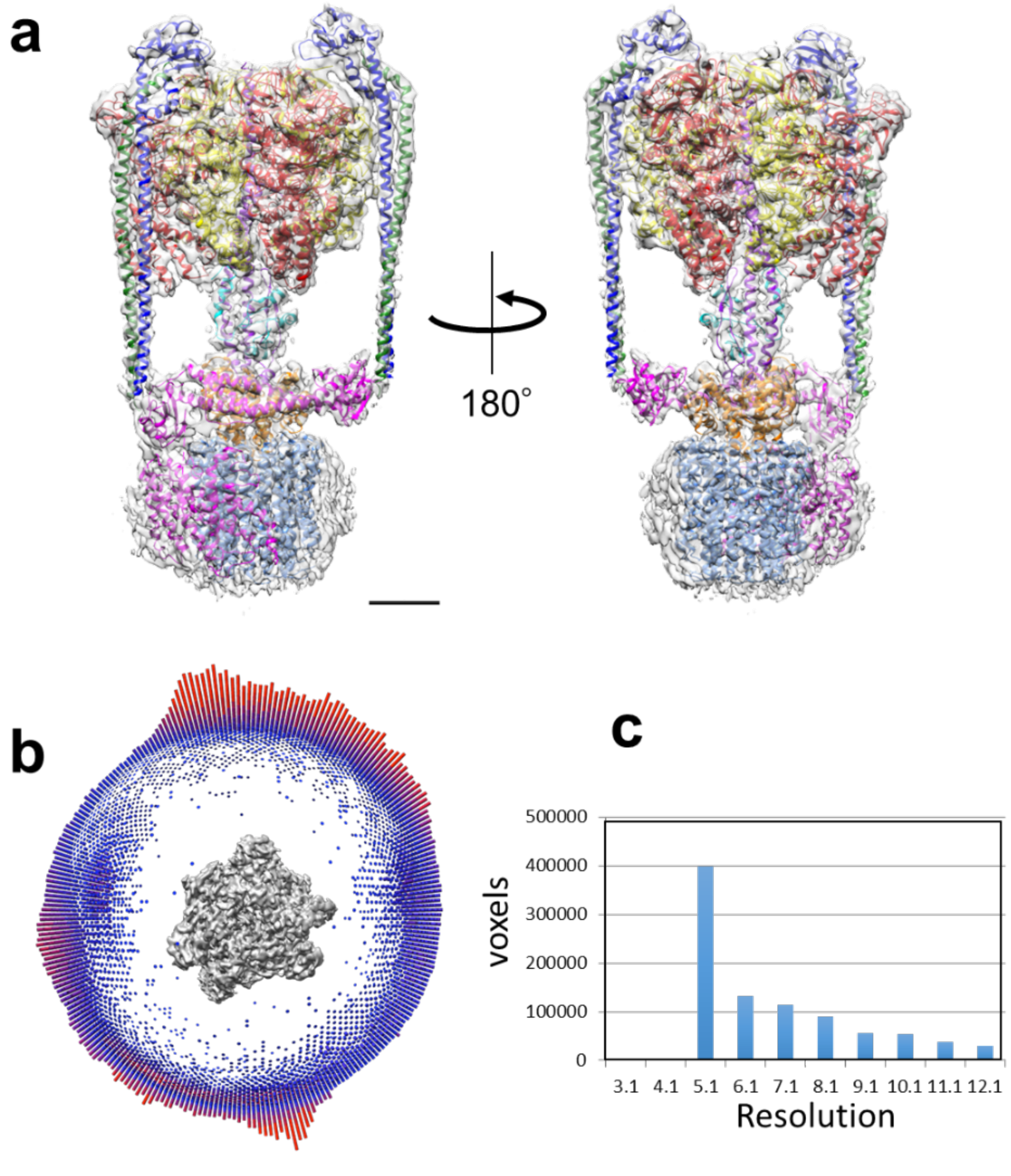

Supplementary figure 10. EM map data of V/A-ATPase state1. **a**, The refined map of state1. Experimental maps are shown in semi-transparent grey. The atomic models of V/A-ATPase are fitted into the maps by using MDFF. Scale bar, 30 Å. **b**, Euler angle distribution in the top view of the map. **c**, Histogram of resolution of voxels.

Supplementary table 1. Data correction for the V/A-ATPase.

	State 1 (Overall)	State 1 (Peripheral domain)	State 2 (Overall)	State 2 (Peripheral domain)	State 3 (Overall)	State 3 (Peripheral domain)
EMDB ID	EMDB-6810	EMDB-6811	EMDB-6812	-	EMDB-6813	-
PDB ID	5Y5X	5Y5Y	5Y5Z	-	5Y60	-
**Data collection and processing**						
Magnification	59K					
Voltage (kV)	300					
Electron exposure (e–/Å^2)	26					
Defocus range (μm)	2.5-3.5					
Pixel size (Å)	1.4					
Symmetry imposed	C1					
Micrograph collected (no.)	4.7K					
Final particle images (no.)	118K	130K	30.8K	34K	13.8K	16K
Map resolution (Å)	5.0	4.7	6.7	5.9	7.5	8.1
FSC threshold						
**Refinement**						
Initial model used (PDB code)	5GAR					
Model resolution (Å)	5.06	4.70	6.90		7.50	
FSC threshold						
Map sharpening *B* factor (Å^2)	-303.38	-253.69	-350.79		-347.60	
Model composition						
Non-hydrogen atoms	44393	32516	44394		44394	
Protein residues	5852	4166	5853		5853	
Ligands	2	2	2		2	
R.m.s. deviations						
Bond lengths (Å)	0.024	0.027	0.024		0.024	
Bond angles (°)	2.65	2.90	2.64		2.79	
Validation						
MolProbity score	2.56	2.92	3.29		3.09	
Clashscore	4.25	6.83	17.0		10.9	
Poor rotamers (%)	7.3	9.2	10.7		11.4	
Ramachandran plot						
Favored (%)	82.8	73.3	77.0		80.3	
Allowed (%)	12.0	17.1	15.4		13.2	
Disallowed (%)	5.2	9.6	7.6		6.5	
