# Supplementary material for: Cryo EM structure of intact rotary H+-ATPase/synthase from Thermus thermophilus
Source: Nat Commun. 2018 Jan 8;9:89. doi: 10.1038/s41467-017-02553-6 (PMC5758568; doi:10.1038/s41467-017-02553-6)
Supplement: Supplementary file 3 — Description of Additional Supplementary Files [file 41467_2017_2553_MOESM3_ESM.pdf]

**Description of Additional Supplementary Files**

File Name: Supplementary Movie 1
Description: A morphing movie among the three states with TM domain of a-subunit fixed when they work as ATP synthase. The order of the picture is state1, 3, 2 and 1. Side view.

File Name: Supplementary Movie 2
Description: A morphing movie of a close-up view of A3B3 domain with N-terminal β-barrel domain of B-subunit fixed when they work as ATP synthase. Side and top view.

File Name: Supplementary Movie 3
Description: A morphing movie of a close-up view of the stalk region of EG and a-NT with TM domain of a-subunit fixed when they work as ATP synthase. Side view.
